# Design of an Inertial-Sensor-Based Data Glove for Hand Function Evaluation

**DOI:** 10.3390/s18051545

**Published:** 2018-05-13

**Authors:** Bor-Shing Lin, I-Jung Lee, Shu-Yu Yang, Yi-Chiang Lo, Junghsi Lee, Jean-Lon Chen

**Affiliations:** 1 Department of Computer Science and Information Engineering, National Taipei University, New Taipei City 23741, Taiwan; bslin@mail.ntpu.edu.tw (B.-S.L.); akino_sumiko@hotmail.com (I.-J.L.); 2 College of Electrical Engineering and Computer Science, National Taipei University, New Taipei City 23741, Taiwan; 3 Department of Physical Medicine and Rehabilitation, Chi-Mei Medical Center, Tainan 71004, Taiwan; yangshuyu1970@gmail.com; 4 Department of Electrical Engineering, Yuan Ze University, Taoyuan City 32003, Taiwan; alexlo0703@gmail.com (Y.-C.L.); eejlee@saturn.yzu.edu.tw (J.L.); 5 Department of Physical Medicine & Rehabilitation, Taoyuan Chang Gung Memorial Hospital, Taoyuan City 333, Taiwan; 6 Center for Healthy and Aging Research, Chang Gung University, Taoyuan City 33302, Taiwan; 7 School of Medicine, Medical College, Chang Gung University, Taoyuan City 33302, Taiwan

**Keywords:** motion capture, data glove, inertial sensor, joint measurement, rehabilitation

## Abstract

Capturing hand motions for hand function evaluations is essential in the medical field. Various data gloves have been developed for rehabilitation and manual dexterity assessments. This study proposed a modular data glove with 9-axis inertial measurement units (IMUs) to obtain static and dynamic parameters during hand function evaluation. A sensor fusion algorithm is used to calculate the range of motion of joints. The data glove is designed to have low cost, easy wearability, and high reliability. Owing to the modular design, the IMU board is independent and extensible and can be used with various microcontrollers to realize more medical applications. This design greatly enhances the stability and maintainability of the glove.

## 1. Introduction

Capturing hand kinematics is necessary in medical applications, such as rehabilitation and hand function evaluation [1,2,3]. By capturing hand kinematics, physicians can accurately determine the recovery progress of stroke patients’ hand. Therefore, a practical and reliable device that can record several hand motion parameters in the rehabilitation field is needed.

Studies on hand kinematics capturing have used noncontact-based or wearable technology [2,3,4,5,6,7,8,9,10,11,12,13]. Noncontact-based approaches mainly use cameras or depth-based cameras to capture hand kinematics and analyze continuous hand movements through image processing [4,6,7]. The main drawbacks of noncontact-based systems are reported in the previous studies [14]. The major problem of noncontact-based systems is that it is restricted by the place where the camera placed and the lighting condition of the environment. Moreover, self-occlusion when the hand is in a non-observed condition also causes the inaccurate kinematics capturing. Therefore, wearable systems are more practical for capturing the hand kinematics precisely without environmental restrictions.

Data gloves are the most popular wearable systems. With technological advancements, various sensors, including mechanical sensors, resistive sensors, optical fiber sensors, and inertial measurement units (IMUs), have been incorporated into data gloves for capturing hand kinematics [2,3,5,8,9,10,11,12,13]. Physicians can observe finger motions through sensors attached on the fingers of the data glove. IMUs are the most practical sensors for data gloves [15] because they are light and can accurately capture slight changes in finger motions. IMUs can also provide original values, such as acceleration, angular velocity, and magnetic field. By applying a sensor fusion algorithm to these original values, the range of motion (ROM) that can be interpreted directly by physicians can be obtained [2,8].

Various data gloves have been proposed thus far. In 2014, Kortier et al. [2] and Moreira et al. [13] independently proposed data gloves with IMUs that contained an accelerometer, gyroscope, and magnetometer. Both data gloves could measure finger motion accurately; however, both used wired transmission and caused restrictions when subjects were performing hand function evaluation tasks. Moreover, these data gloves did not have a modular design. In 2016, Choi et al. proposed a low-cost data glove with 9-axis sensors [11]. However, they only conducted a static validation; a dynamic validation is required in the medical field. Without dynamic validation, the ROM measurement accuracy when the subject was conducting hand evaluation tasks could not be determined. In 2017, Fang et al. developed a data glove with an attitude measurement algorithm [8]. The authors conducted both static and dynamic validation to verify the accuracy of the algorithm; however, the hardware design of this data glove was not modular, and therefore, the glove could not be used if one of its sensors was damaged. 

Therefore, although many studies have focused on data gloves, aspects such as the transmission method and modular design provide scope for improvement. To modify the design and avoid the drawbacks of previous studies, a hand function assessment system using a data glove with 6-axis IMUs was proposed in 2017 [16]. However, the sensor used in this study contained only an accelerometer and gyroscope and could only measure the ROM in a specific setting. Owing to the lack of a magnetometer, the error increases with time and causes inaccuracy in the heading angle. In addition, the system used Bluetooth as the transmission protocol. The Bluetooth module had a baud rate of 115,200 bps; this was insufficient for the large amount of data obtained from the 16 IMUs, thus reducing the system reliability. Moreover, there was only one IMU on the back of the hand, and therefore, the ROM of the metacarpophalangeal joints of the thumb and little finger could not be captured accurately.

To eliminate the aforementioned drawbacks of previous studies, a modular data glove with 9-axis IMU sensors has been presented to provide reliable acceleration, angular velocity, and ROM as the parameters of manual dexterity to physicians. The proposed data glove has a modular design that increases its expandability. Each module can operate independently, for instance, the mainboard provides various common data transmission protocols that can combine different types of biomedical sensors in the future. In addition, to enhance the independence of the sensor part, the IMU is soldered on a traditional printed circuit board (PCB) to form an IMU board. And the IMU board is connected with the flexible printed circuit board (FPCB) with a pin header. This novel approach not only enhances the maintainability of the data glove but also provides the function of communicating with various microcontrollers.

## 2. System Architecture and Hardware Design

### 2.1. System Overview

This study aims to implement a data glove with a modular design. This glove can be worn easily and can collect motion parameters while the subject is performing hand function assessment tasks. After receiving motion parameters from the data glove, the sensor fusion algorithm in the software calculates and converts the raw data into a ROM that can be interpreted directly by physicians. The design of the data glove includes the hardware and software design.

**Figure 1:**
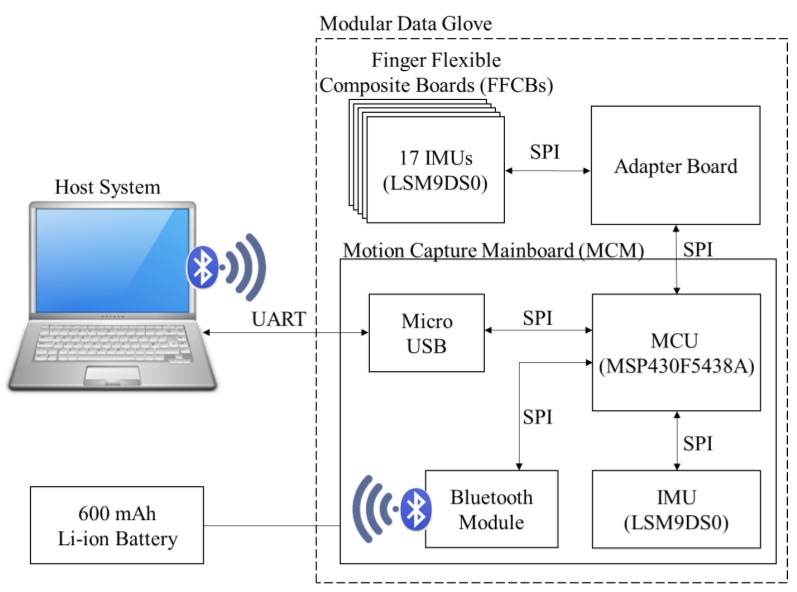
System architecture of data glove system.

The hardware design includes three parts: (1) motion capture mainboard (MCM); (2) finger flexible composite board (FFCB) and IMU sensor board (IMU-SB); and (3) adapter board and host system. The MCM is powered by a Li-ion polymer battery and is worn on the subject’s forearm. The adapter board is the bridge between the MCM and the FFCB. A flat flexible cable is used to connect the MCM and the adapter board. Five FFCBs are attached on the five fingers of the data glove, and they are connected to the adapter board. Figure 1 shows the system architecture. MCM collects the raw data from the 17 IMUs on the five FFCBs and one IMU on the MCM and sends it to the host system via Bluetooth.

**Figure 2:**
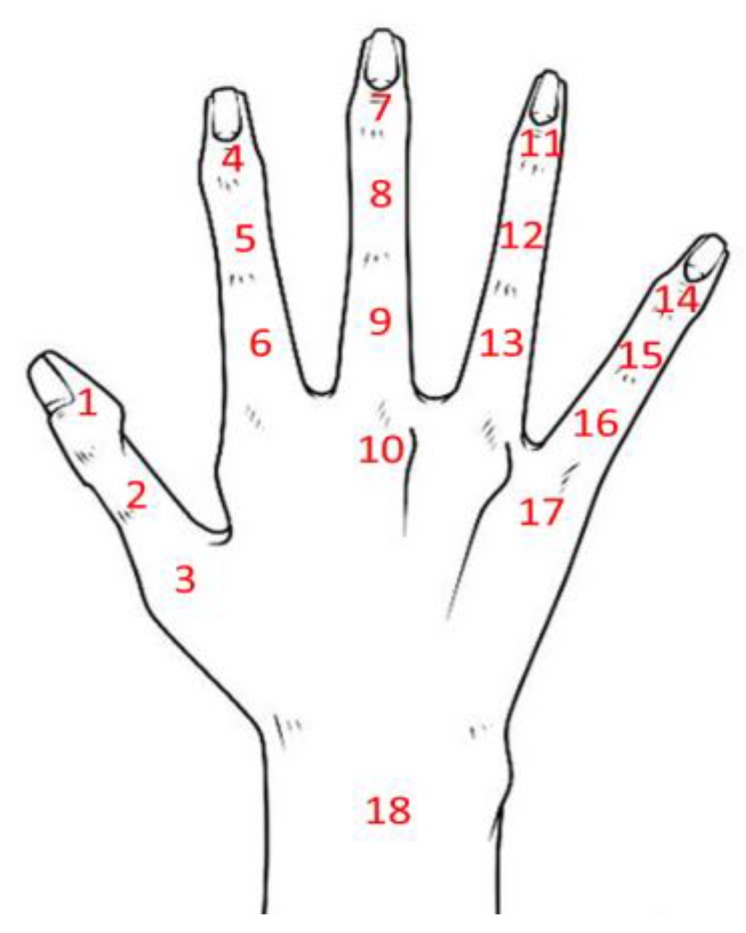
IMU positions on the data glove.

Figure 2 show the positions of the 18 IMUs in the proposed data glove. In Figure 2, positions 1–17 indicate sensor positions, and position 18 indicates the sensor on the forearm. To measure the ROM of the MCP joints more accurately, the sensors at positions 3, 10, 17 and 18 are used to reconstruct and capture the finger and hand motions in the three-dimensional space. The ROM of the MCP joint of the thumb can be calculated using the data from sensors 2 and 3. The ROM of the MCP joint of the index, middle, and ring fingers can be calculated using the data from sensors 6, 9 and 13. The ROM of the MCP joint of the little finger can be obtained using the data from sensors 16 and 17. Sensor 18, which is on the MCM, is used to collect the motion parameters of the forearm. The ROM of the wrist can be calculated using sensors 10 and 18.

**Figure 3:**
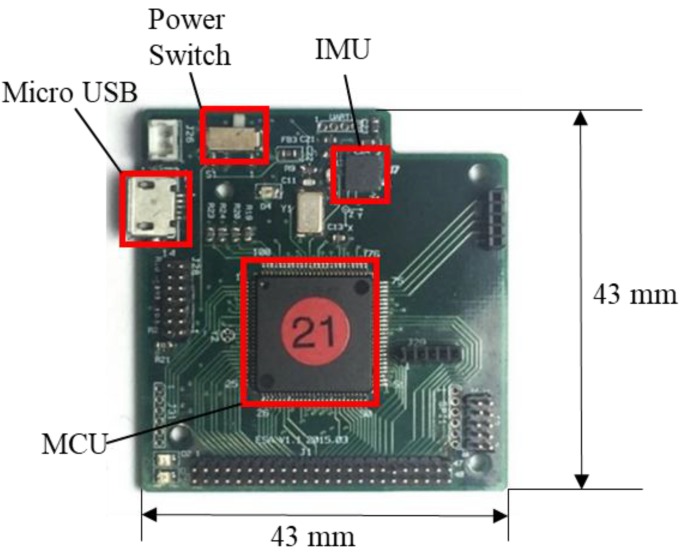
MCM components.

Figure 3 shows the MCM, which is custom-made as the main core of the system. The size of the MCM is 43 mm × 43 mm. The MCM contains a microcontroller, an IMU, a Bluetooth module, and a 600-mAh Li-ion polymer battery as the power source. The microcontroller (MSP430F5438A, Texas Instruments, Dallas, TX, USA), which is the main component of the data glove, collects data via serial peripheral interface (SPI) bus, encapsulates the data into packets, and sends the packets to the host system via Bluetooth. Three extensible interfaces for the universal asynchronous receiver/transmitter (UART), SPI, and analog-to-digital converter are reserved for integrating other biomedical sensors in the future.

**Figure 4:**
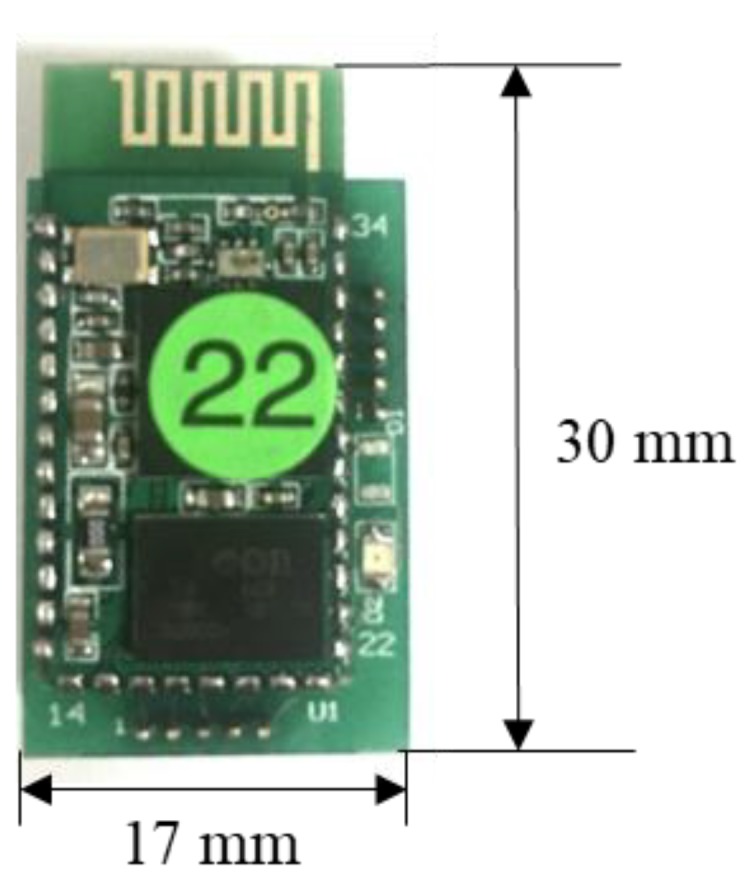
Bluetooth module and wireless adapter board.

HL-MD08R (Hotlife Electronic Technology Co., Ltd., Taipei, Taiwan) was used as the transmitting module of the data glove. Figure 4 shows the HL-MD08R module. This device has a maximum baud rate of 921,600 bps; this is sufficient for transmitting packets from the data glove. In addition, HL-MD08R is soldered on the wireless adapter board, and this board is plugged into the UART port of the MCM. The UART interface has an extensible port that can support various wireless communicating modules in the future.

A 9-axis IMU (LSM9DS0, STMicroelectronics, Geneva, Switzerland) was used for motion capture in the proposed data glove. The IMU contains a 3-axis magnetometer, a 3-axis gyroscope, and a 3-axis accelerometer, and it can provide the 3-axis acceleration, 3-axis angular velocity, and 3-axis magnetic field. LSM9DS0 was soldered on a 10-mm × 10-mm IMU-SB. The IMU-SB has a modular design, and it can capture finger motions and independently operate with other types of microcontrollers to enable novel applications.

### 2.2. Mechanical Design of Data Glove

**Figure 5:**
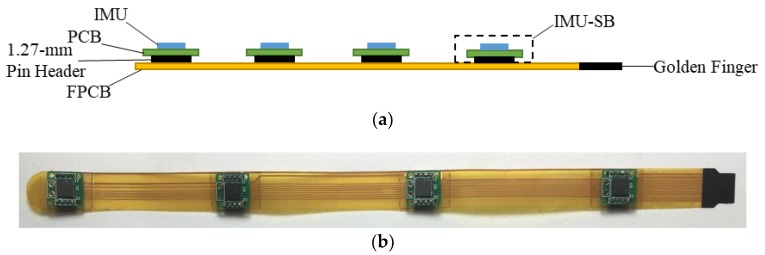
(**a**) Side view of FFCB and (**b**) FFCB.

The modular design of the proposed data glove enhances the extensibility of its components, especially in terms of FFCB design. Figure 5a shows a side view of the FFCB design. The traditional method is to solder the IMU directly on an FPCB [12]; however, this approach increases the risk of split solder and instability of the IMU signal. In this study, the IMU was soldered on the traditional PCB to form an IMU-SB, and then, the IMU-SB was combined with FPCB to form the FFCB. This design enhances the stability and reliability of IMU signals. The failure rate of the IMUs can also be reduced by using this design. The connection between the MCU and the IMUs relies on the FPCB circuit. The power lines and signal lines of the circuits are on different layers to avoid split solder in the FPCB. The proposed data glove used a 1.27-mm pin header to connect the IMU-SB and the FPCB. If an IMU or a FPCB is broken, only the broken component is required to be changed. This design enhances the maintainability and extends the lifetime of the data glove. A golden finger is used on the tail of each FFCB, and it can be quickly and directly connected to the flip lock connector on the adapter board. This design reduces the number of connecting points between each component and avoids unstable signals that could cause incorrect data acquisition. At most four IMU-SBs can be mounted on each FFCB depending on the application. Figure 5b shows the FFCB obtained by combining the FPCB and IMU-SB.

**Figure 6:**
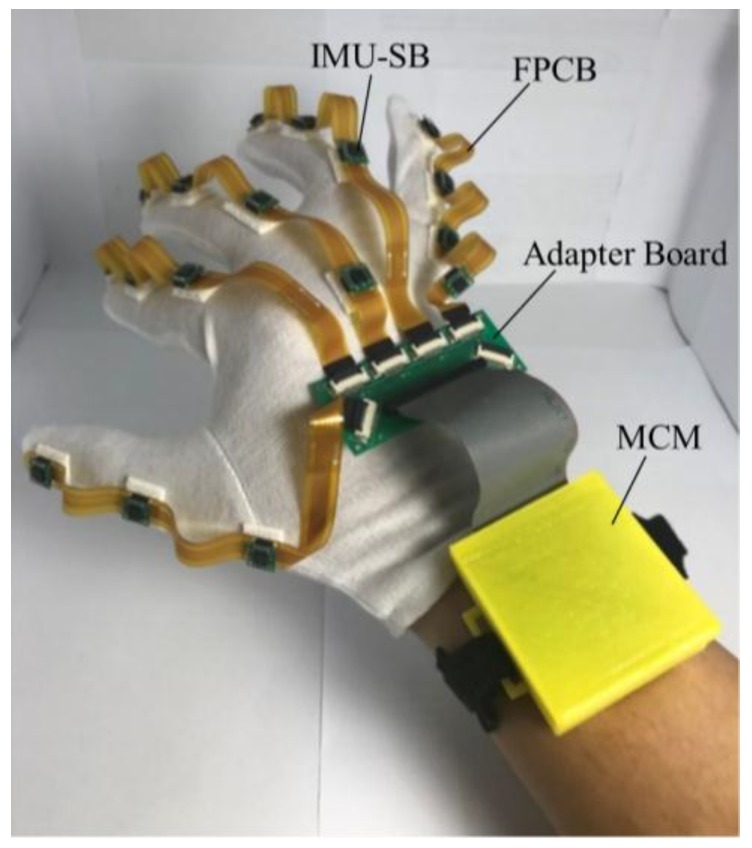
Prototype of the data glove.

Figure 6 shows the prototype of the data glove. The shell of the MCM is created using a 3D printer. Velcro is used to fasten the MCM to the forearm. The FFCBs and the adapter board are fastened to a cotton glove using 3M picture hanging strips (Command™ Narrow Picture Hanging Strips, 3M Company, St. Paul, MN, USA), which is firmer than traditional Velcro and can be removed easily.

## 3. Software Design

### 3.1. Flowchart of Software

**Figure 7:**
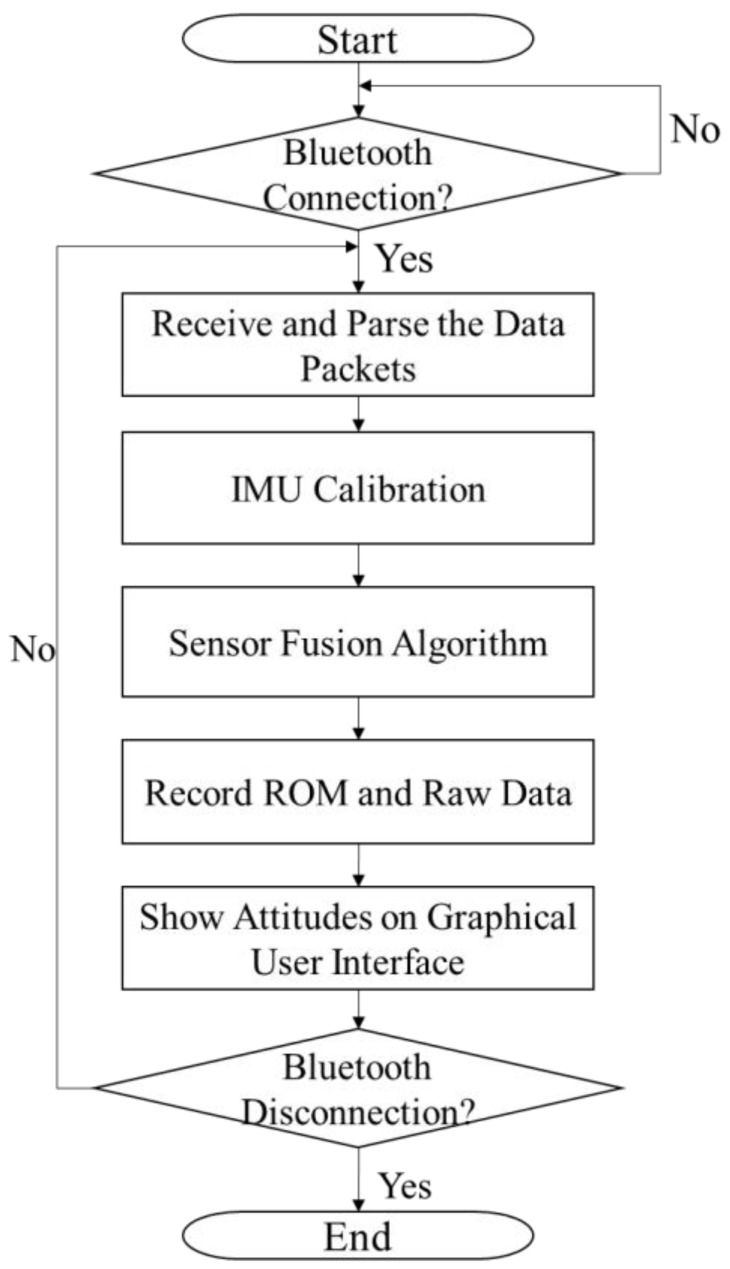
Flowchart of program on host system.

The program on the host system was developed in C#. Figure 7 shows its flowchart. When the program begins, the graphical user interface (GUI) is created and initialized. Next, the COM port of the Bluetooth module is selected by the user, and then, a connection is established between the program and the data glove. After connecting successfully, the program on the host system starts to receive packets from the data glove and conducts sensor calibration and attitude calculation. The attitude is converted to the ROM, shown on the GUI, and recorded.

### 3.2. Sensor Calibration

To ensure the IMU sensor performance will meet its specifications, IMU calibration is necessary before executing the sensor fusion algorithm. In this study, the IMU is placed on the table to calibrate the accelerometer and gyroscope. The accelerometer offset ${\mathrm{Acc}}_{\mathrm{offset}}$ and gyroscope offset ${\mathrm{Gyro}}_{\mathrm{offset}}$ are both obtained by averaging 1000 raw data measurements from the accelerometer and gyroscope, respectively.

**Figure 8:**
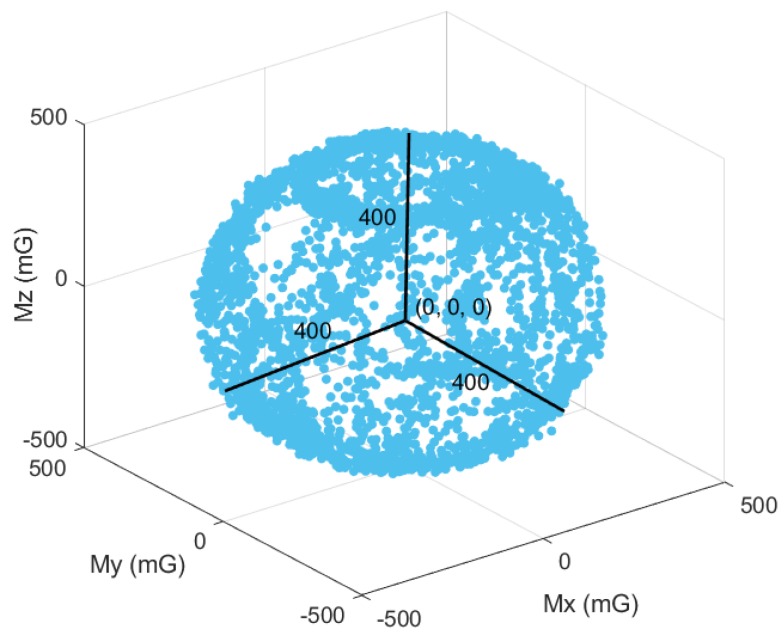
Well-calibrated raw data from the magnetometer. The center of the distribution is at (0, 0, 0) and the radius is 400 mG.

The magnetometer is calibrated differently from the accelerometer and gyroscope. The magnetometer is required to conduct an 8-shaped rotation to find the maximum and minimum values on each axis. ${\mathrm{Mag}}_{\max}$ and ${\mathrm{Mag}}_{\min}$ are the maximum and minimum values, respectively, of the axis. The magnetometer offset ${\mathrm{Mag}}_{\mathrm{offset}}$ is the average of ${\mathrm{Mag}}_{\max}$ and ${\mathrm{Mag}}_{\min}$ [17]. Figure 8 shows well-calibrated raw data from the magnetometer. After calibration, the center of the distribution of the raw data will be very close to (0, 0, 0). It means that the hard iron calibration had finished. It will also become a spherical distribution with radius of 400 mG, meaning that the soft iron calibration had finished.

After obtaining the accelerometer, gyroscope, and magnetometer offsets, the raw data from these three sensors are adjusted using these offsets, as shown in (1)–(3). The calibrated data, ${\mathrm{Acc}}^{\prime }$, ${\mathrm{Gyro}}^{\prime }$, and ${\mathrm{Mag}}^{\prime }$, are inputted into the sensor fusion algorithm to obtain the attitudes:

(1) $${\mathrm{Acc}}^{\prime }=\;\mathrm{Acc}-{\mathrm{Acc}}_{\mathrm{offset}}$$

(2) $${\mathrm{Gyro}}^{\prime }=\mathrm{Gyro}-{\mathrm{Gyro}}_{\mathrm{offset}}$$

(3) $${\mathrm{Mag}}^{\prime }=\mathrm{Mag}-{\mathrm{Mag}}_{\mathrm{offset}}$$

### 3.3. Sensor Fusion Algorithm

Several sensor fusion approaches have been proposed for various applications [18,19,20,21,22,23,24]. Kalman-based filters are the most common approaches in the previous studies; however, Kalman-filter is not always suitable for motion capturing [23]. It is time-consuming to use Kalman filter to measure the attitude of multiple sensors because of the complex computation of the algorithm. Therefore, our study used Madgwick’s sensor fusion algorithm [24], which is more efficient and requires less computing time. The attitude obtained from the gyroscope and that from the accelerometer and magnetometer were combined by the algorithm to obtain the final attitude.

The attitude obtained from the gyroscope is updated as shown in (4). The quaternion q represents the attitude of the IMU. $q_{t}$ is the attitude at the current time. $q_{t-1}$ is the attitude at the previous time. $\omega_{t-1}$ is the angular velocity at the previous time. $\Delta_{t}$ is the time difference between the previous time and the current time:

(4) $$q_{t}=q_{t-1}+\frac{1}{2}q_{t-1}\times \omega_{t-1}\Delta_{t-1}$$

The attitudes obtained from the accelerometer and magnetometer are updated as shown in (5). The gradient descent method is used to find the optimal solution of the attitude. μ is the step size of the gradient descent. *f* is the objective function, which represents the error between $q_{t-1}$ and the output of accelerometer and magnetometer. ∇f is the objective function gradient which is defined by the measurement of accelerometer and magnetometer at time *t*:

(5) $$q_{t}=q_{t-1}-\mu \frac{\nabla f}{\left\|\nabla f\right\|}$$

Finally, Equations (4) and (5) are combined to obtain the final attitude, as given by (6). β is the gyroscope error. This equation optimizes the solution along with the direction which the error decreased. After optimization, the final attitude is obtained.

(6) $$q_{t}=q_{t-1}+\frac{1}{2}q_{t-1}\times \omega_{t-1}\Delta_{t-1}-\beta \frac{\nabla f}{\left\|\nabla f\right\|}\Delta_{t-1}$$

**Figure 9:**
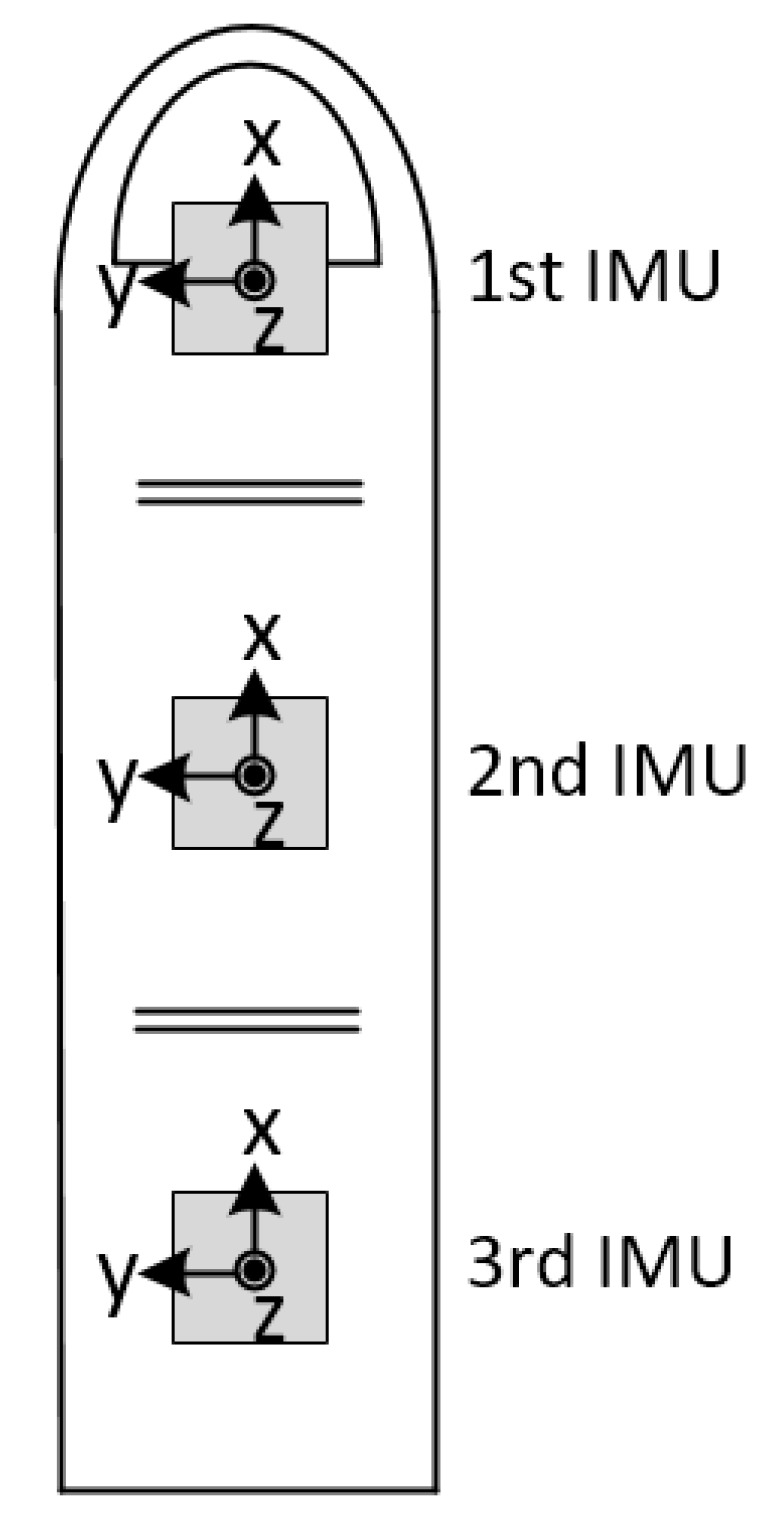
Direction of IMUs.

After obtaining the quaternion of the current attitude by using the above algorithm, the ROM is calculated. Figure 9 shows the 3-axis direction of the IMU. The positive direction of the IMU’s *x*-axis is the same as the direction in which the finger points. The positive direction of the IMU’s *y*-axis is perpendicular to the direction in which the finger points. The positive direction of the IMU’s *z*-axis is perpendicular to and points out of the finger plane. The ROM is calculated by converting the quaternions of two adjacent IMUs to Euler angles and by obtaining the difference between the *y*-axis angles. The current attitude $\left(q_{1},\;q_{2},\;q_{3},\;q_{4}\right)$ is converted to the Euler angle of the IMU’s *y*-axis as shown in (7):

(6) $$\theta =-\sin^{-1}\left(2q_{2}q_{4}+2q_{1}q_{3}\right)$$

**Figure 10:**
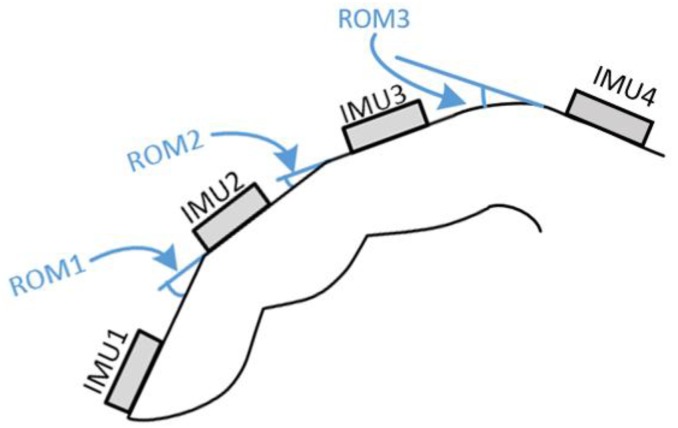
Position of ROMs and IMUs.

In this research, at most four IMUs can be mounted on the FFCB. With the IMU on the back of the hand, three ROMs can be calculated. Figure 10 shows the position of the ROMs and IMUs. ROM1, obtained from IMU1 and IMU2, is the ROM of the distal interphalangeal joint. ROM2, obtained from IMU2 and IMU3, is the ROM of the proximal interphalangeal joint. ROM3, obtained from IMU3 and IMU4, is the ROM of the metacarpophalangeal joint.

## 4. Verification of Data Gloves

### 4.1. Verification of Raw Data

**Figure 11:**
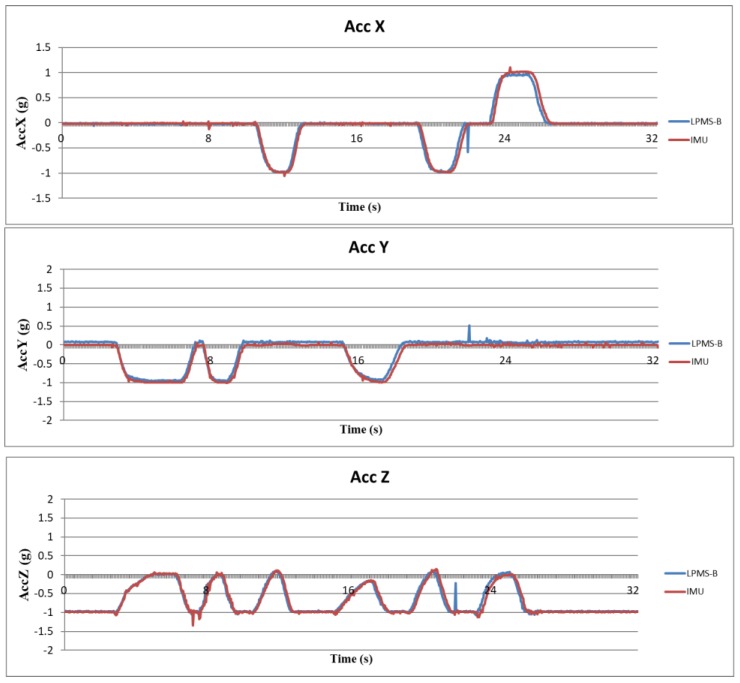
Validation results of accelerometer data.

**Figure 12:**
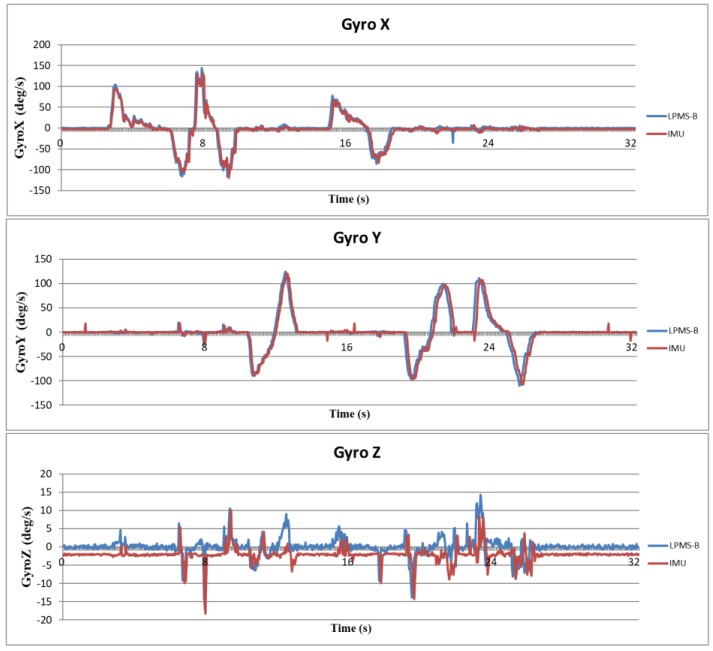
Verification results of gyro data.

**Figure 13:**
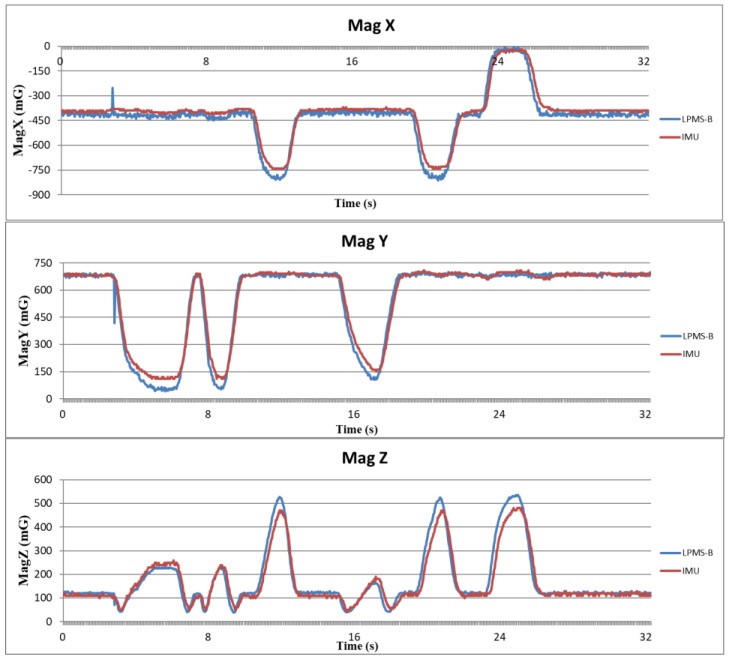
Verification results of magnetometer data.

Because the attitude angle is calculated from the acceleration, angular velocity, and magnetic orientation, it is necessary to verify that the data obtained from the IMU is correct before calculating the attitude angle. In this study, a trusted IMU device (LPMS-B, LP-Research, Tokyo, Japan) was used to compare the raw data with the sensors used in this study. LPMS-B is an accepted standard in many laboratories [25,26], and its raw data output is reliable. The raw data is verified by fixing the MCM and LPMS-B on the same board and swinging the board randomly and continuously. The sampling rates of both the devices were set to 25 Hz. The scales of both the two sensors were set to ±4 g for accelerometer, ±8 g for gyroscope, and ±400 μT for the magnetometer. The host receives raw data from the MCM and LPMS-B simultaneously, thus enabling the verification of whether the data are consistent. Figure 11, Figure 12 and Figure 13 show the analysis results for the accelerometer, gyroscope, and magnetometer, respectively. Root mean square error (RMSE) is used to quantify the error. For accelerometer, RMSEs of *x*-axis, *y*-axis, and *z*-axis are respectively $6.78\times 10^{-2}$ g, $8.80\times 10^{-2}$ g and $8.34\times 10^{-2}$ g. For gyroscope, RMSEs of *x*-axis, *y*-axis and *z*-axis are 10.29 deg/s, 11.86 deg/s, 3.37 deg/s, respectively. For magnetometer, because the distortion of magnetic field of each sensor is different, the magnetic field of IMU was shifted to the same level as LPMS-B to obtain RMSEs. RMSEs of *x*-axis, *y*-axis, and *z*-axis are 36.95 mG, 32.40 mG, and 32.56 mG, respectively. The results show that the raw data outputted from the IMUs of our proposed system are accurate and have high level of confidence.

**Figure 14:**
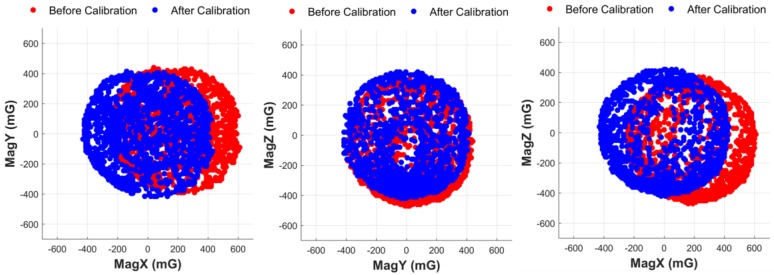
The data of magnetometer before and after calibration.

To ensure the magnetometer is well-calibrated, the distortions of *x*-axis, *y*-axis and *z*-axis of magnetometer have to be under an acceptable level. The sensor is rotated in repeated figure-eight path to calibrate, and then measuring and recording the distortion. Figure 14 shows the data of magnetometer before and after calibration. The figure plots the data on every two axes to show the result more clearly. The original data before calibration is plotted as red dots and the calibrated data is plotted as blue dots. The distortions of the original raw data are 180 mG, 25 mG, and −50 mG on *x*-axis, *y*-axis, and *z*-axis, respectively. Figure 14 also shows that by applying the calibrating method in Section 3.2, the centers of the calibrated data are all at (0, 0, 0) and the radius of the spheres are 400 mG, showing that the calibrated data of magnetometer is reliable. 

### 4.2. Verification of Static Finger Angles

**Figure 15:**
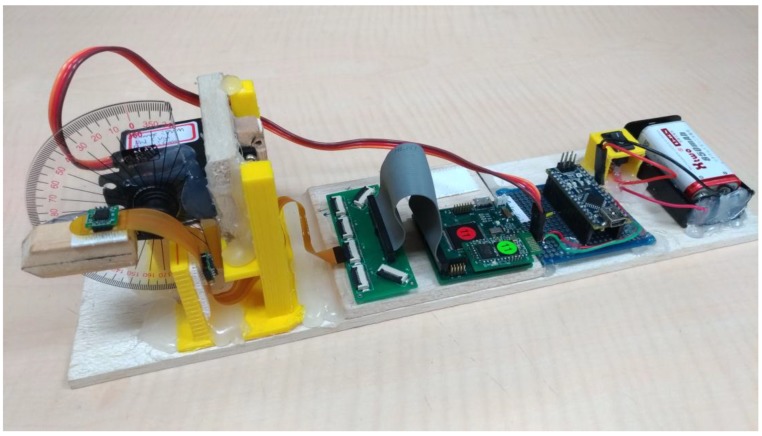
Self-made measurement platform for measuring finger angle.

In this study, a self-made finger angle measurement platform is used for verifying the static finger angle, as shown in Figure 15. A folding ruler with an angle ruler is combined with a wood stick and wood block to form the measuring platform, and FFCB is fixed on the platform using 3M picture hanging strips. The measuring platform can then simulate the bending of the first knuckle to observe the angle change of the finger ROM1 in Figure 10.

For verifying ROM1, we used bending angles of 0°, 30°, 60°, 90°, and 120° and averaged 1000 measurements at each angle to verify the angle accuracy. Table 1 shows the average of the measured angles calculated using the software and RMSE obtained by comparing the average of the measured angles with the actual angles. The RMSE are all less than 0.2°, indicating that the sensor fusion algorithm used in our proposed system for calculating static ROM1 is stable and reaches a very good standard.

### 4.3. Verification of Dynamic Finger Angles

**Figure 16:**
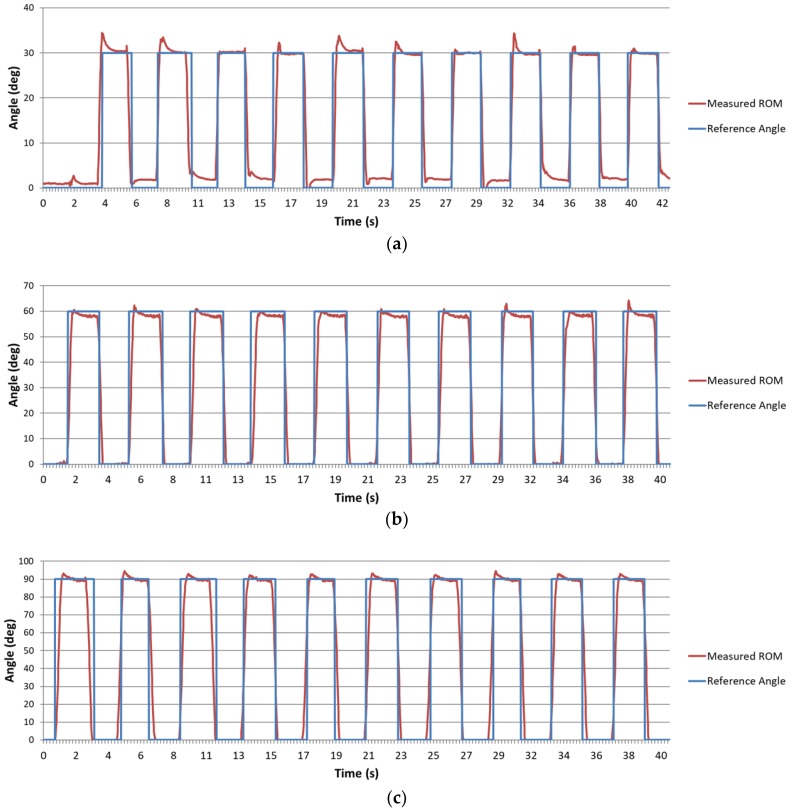
Self-made measurement platform for measuring finger angle. The angles are set to change in different intervals: (**a**) interval 0°–30°; (**b**) interval 0°–60°; and (**c**) interval 0°–90°.

Dynamic verification uses the same measurement tools as static verification. The test method uses a servo motor to control a wood stick swinging back and forth within an angle interval. The servomotor (MG995, TowerPro, Taipei, Taiwan) was used for the dynamic verification. A program developed in Arduino was used to control the servomotor to the specific angle with pulse width modulation (PWM). In this experiment, three test intervals were set to 0°–30°, 0°–60°, and 0°–90°. The ROM changes every 2 s and recorded angles are compared with the reference angles given by the servomotor. This verification method is used to verify whether ROM1 calculated using the algorithm can maintain its accuracy and stability when the IMU sensors of the FFCB move in different ranges. Figure 16 shows the ROM1 waveforms calculated from the algorithm used for dynamic verification. This figure shows that the angles oscillate regularly in these three intervals; Table 2 shows the error percentages. The mean errors are 0.91°, −2.30°, −0.82° for dynamic angle ranges of 30°, 60°, and 90°, respectively.

### 4.4. Verification of Stability of Angle Measurement

**Figure 17:**
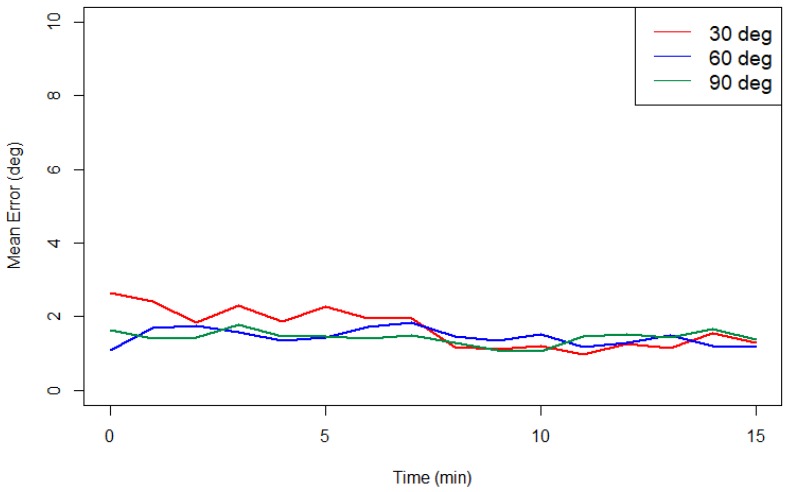
The mean error of the static angle for each minute.

To ensure the stability of the fusion algorithm, an experiment is conducted to verify the stability of the algorithm of ROM measurement. Reference angle is set on 30°, 60°, and 90° with servomotor and the measured ROM were recorded for 15 min. The experiment was only conducted in static situation to eliminate other potential factors during dynamic verification. Figure 17 presents the mean error of the measured ROM in every minute. The mean errors are all less than 3° during 15 min, showing that the stability of the algorithm.

### 4.5. Real-Time Visual Operation

**Figure 18:**
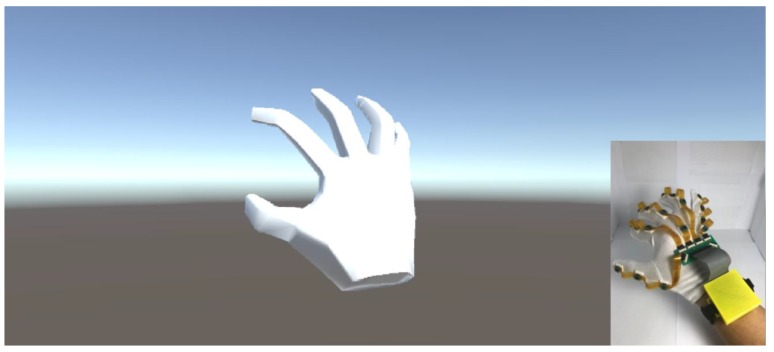
The real-time Unity program for visualizing the hand’s movement.

To ensure the relative posture of each sensor, a real-time visualized Unity program was developed. The screenshot of the visualized program and posture of the data glove are depicted in Figure 18. Implementing visualization of the data from the data glove can help to ensure whether the system is calibrated well. If all of the sensors are calibrated well, the virtual hand will move perfectly and simultaneously with the real hand.

## 5. Discussion

This study proposed an inertial-sensor-based data glove. The glove mechanism was designed to have low cost, modularity, high expansibility, and complete system integration.

To verify the reliability and stability of the data gloves, three verification experiments were designed and conducted. First, the raw data were verified. The results showed high correlation between LPMS-B and the 9-axis IMUs used in this study; therefore, the output data of this IMU sensor can be trusted. Second, the static angle was verified. The results showed that the RMSE were all less than 1° at any static angle, indicating that the ROM calculated by the sensor fusion algorithm has high accuracy in the static state. Third, the data gloves were verified under dynamic conditions because they must be able to record the parameters of the finger’s continuous motion in most applications. The results show that the proposed data glove has the mean error of angle under ±3° in motion. This error might be attributed to the test tool itself, resulting in the inability to accurately change back and forth. Moreover, the reference angles can only be reported every 20 ms when the angle is at a certain static angle, but the transition state of the servomotor cannot be reported because of the PWM requires at least 500 ms to change the angle. However, in rehabilitation activities or hand function assessments, the mean error of angle under ±3° is acceptable to the physicians while the patients are conducting the rehabilitation tasks [27,28]. Therefore, our proposed data glove remains applicable in the medical domain. The last experiment is the verification of the stability. The result shows that the fusion algorithm is stable during a long period of time.

This designed data gloves are modular. The FPCB and IMU-SB are connected through a pin header to enable IMU-SBs to be replaced easily or for the number of IMU-SBs to be increased or decreased easily. However, this design remains inconvenient from the viewpoint of disassembly. Therefore, thinner and smaller PCBs and circuit board connectors will be used in the future, and these will be embedded in the gloves as a spring buckle. The integrated design of the IMU-SB, FPCB, and data glove should also be considered to enhance the stability and portability.

The microprocessor of the MCM currently simply collects IMU data and transmits it to the host system through wired or wireless transmission for parameter calculation. The packets transmitted by the glove contain the original IMU data that is very large. The data sampling rate of the algorithm is limited to 50 Hz owing to the transmission bandwidth. Therefore, the algorithm can be implemented in firmware in the future, and the original data output can be changed to quaternion attitude data to greatly reduce the amount of data and the load on the host system software. Another option is to increase the transmission bandwidth by using Wi-Fi transmission, instead of Bluetooth transmission.

Table 3 compares this study with several previous studies on data gloves. Kortier et al. developed data gloves that accurately captured dynamic changes in finger motions [2]. However, this glove could only transmit data through a USB interface, thus limiting its use. Choi et al. developed a data glove and algorithm for calculating the attitude angle effectively [11]. However, they did not provide the ROM, and they only performed static verification for their data glove. Fang et al. proposed a data glove for capturing finger motions and hand dynamics and displaying them on a screen [8]. However, this glove had many wires, leaving it susceptible to damage. None of the aforementioned three studies have used a modular design; therefore, if any damage was difficult to repair, the glove components could not be used for other applications. The data glove proposed in this study overcomes these problems by adopting a modular design, soldering the IMU to the traditional circuit board, and then combining with the FPCB via the pin header to form an FFCB. Because the IMU-SB is separated from the FPCB, the IMU can stably output data, and it does not suffer damage easily. If the IMU-SB or FPCB is damaged, it can be replaced independently. This design can extend the life of the various components of the data glove, and it enables the number of IMU sensors to be adjusted freely in the future. The independent design of the IMU-SB can also be combined with other microprocessors for extended applications. In addition, the MCM is designed independently and has a different communication interface on the board; therefore, it can operate independently or in combination with other sensors. This design of the data glove enables it to capture finger motions and greatly increases its future scalability.

## 6. Conclusions

This study proposed a data glove design for motion capture with eighteen 9-axis IMU sensors on the fingers, wrist, and forearm for measuring hand movement parameters. In addition to the acceleration, velocity, and rotation of hand movements, accurate bending angles of the knuckles of the fingers and the wrist can also be measured and calculated through attitude fusion algorithms. This study also conducted a complete verification of this data glove. The results of raw data, static, and dynamic verification, stability verification show that our proposed data glove has good accuracy and stability.

The proposed data glove has a modular design in which the MCM and the IMU-SB can both be used independently. This enables components to be replaced easily if they are damaged and enables various microprocessors to be combined with the system to extend its applications easily. The MCM also has an expansion interface through which various biomedical signal sensors can be combined in the future for more medical research applications.

In the future, clinical tests will be conducted, and the parameters obtained using this data glove will be correlated with physicians’ existing scales to enable physicians to more accurately analyze patients’ rehabilitation and hand function. A more automated magnetometer calibration algorithm could be added to the system to make the proposed data glove more suitable for use in hospital environments.

## Figures and Tables

**Table 1:** Verification results of static angles.

Reference Angle (°)	0	30	60	90	120
Average of Measured Angles (°)	0.09	30.04	60.17	88.37	116.47
RMSE (°)	0.15	0.05	0.10	0.19	0.09

**Table 2:** Dynamic verification errors.

Actual Swinging Angle Range (°)	30	60	90
Mean Error (°)	0.91	−2.30	−0.82

**Table 3:** Comparison of our proposed data glove and other data gloves.

System	Kortier et al. [2]	Choi et al. [11]	Fang et al. [8]	Proposed System
Transmission interface	USB	Bluetooth	Bluetooth	USB/Bluetooth
Validation	Dynamic	Static	Dynamic	Raw data, Static, Dynamic
Easy to wear	No	Yes	Yes	Yes
Modular design	No	No	No	Yes
Maintainability	No	No	No	Yes
Extensibility	No	No	No	Yes
